# Good Epidemiologic Practice in Retinitis Pigmentosa: From Phenotyping to Biobanking

**DOI:** 10.2174/138920211795860071

**Published:** 2011-06

**Authors:** Marzio Chizzolini, Alessandro Galan, Elisabeth Milan, Adolfo Sebastiani, Ciro Costagliola, Francesco Parmeggiani

**Affiliations:** 1 Center for Retinitis Pigmentosa of Veneto Region, Camposampiero Hospital, ULSS 15 Alta Padovana, Camposampiero, Italy; 2 Eye Clinic, S. Antonio Hospital, ULSS 16 Padova, Padova, Italy; 3 Department of Ophthalmology, University of Ferrara, Ferrara, Italy; 4 Department of Health Sciences, University of Molise, Campobasso, Italy

**Keywords:** Inherited retinal dystrophies, retinitis pigmentosa, clinical phenotyping, genetic counseling, biobanking, epidemiology.

## Abstract

Inherited retinal dystrophies, such as retinitis pigmentosa (RP), include a group of relatively rare hereditary diseases caused by mutations in genes that code for proteins involved in the maintenance and function of the photoreceptor cells (cones and rods). The different forms of RP consist of progressive neurodegenerative disorders which are generally related to various and severe limitations of visual performances. In the course of typical RP (rod-cone dystrophy), the affected individuals first experience night-blindness and/or visual field constriction (secondary to rod dysfunctions), followed by variable alterations of the central vision (due to cone damages). On the other hand, during the atypical form of RP (cone-rod dystrophy), the cone’s functionalities are prevalently disrupted in comparison with the rod’s ones. The basic diagnosis of RP relies upon the documentation of unremitting loss in photoreceptor activity by electroretinogram and/or visual field testing. The prevalence of all RP typologies is variably reported in about one case for each 3000-5000 individuals, with a total of about two millions of affected persons worldwide. The inherited retinal dystrophies are sometimes the epiphenomenon of a complex framework (syndromic RP), but more often they represent an isolated disorder (about 85-90 % of cases). Although 200 causative RP mutations have been hitherto detected in more than 100 different genes, the molecular defect is identifiable in just about the 50% of the analyzed patients with RP. Not only the RP genotypes are very heterogeneous, but also the patients with the same mutation can be affected by different phenotypic manifestations. RP can be inherited as autosomal dominant, autosomal recessive or X-linked trait, and many sporadic forms are diagnosed in patients with no affected relatives. Dissecting the clinico-genetic complexity of RP has become an attainable objective by means of large-scale research projects, in which the collaboration between ophthalmologists, geneticists, and epidemiologists becomes a crucial aspect. In the present review, the main issues regarding clinical phenotyping and epidemiologic criticisms of RP are focused, especially highlighting the importance of both standardization of the diagnostic protocols and appropriateness of the disease’s registration systems.

## INTRODUCTION

The most frequent hereditary disorders of the posterior segment of the eye are identifiable in the all-encompassing taxonomic terminology of “inherited retinal dystrophies” which are also habitually, even if, imprecisely named retinitis pigmentosa (RP) [1-6]. Despite the relatively high-rate of occurrence of this heterogeneous pathologic category, whose prevalence is variable reported in 1 case for each 3000-5000 individuals [7-23] but can arrives to about 1:2400 or more within close ethnic group knitted by blood relationship or the same kinship [24, 25], the various RP forms are labeled as rare diseases. Rare diseases, including those of genetic origin such as RPs, are life-threatening or chronically debilitating diseases which are of such low prevalence that special combined efforts are needed to address them. However, there is no single, unequivocal, and universally-accepted definition of rare diseases. Some designations are based exclusively on the number of people living with a disease, and other categorizations involve different factors, such as the existence of adequate treatments or the disease’s severity. Similarly, the definitions used in the medical literature or by the Health Systems are again extremely various and, ranging from 1:1000 to 1:200000 in consideration of the disease’s prevalence among general population [26].

The numerous typologies of RP represent the most complex diseases of the eye. In fact, from long-time and from time to time, the scientific literature on RPs has reported the clinical heterogeneity, the phenotypic inter- or intrafamilial variability, and the large genotypic multiplicity which becomes especially evident examining different ethnic clusters [6-25, 27-42]. By means of an extreme cataloging approach manner, to obtain the maximal simplification of an extraordinarily intricate classification, but also knowing that in this way many chances to consider genotype-phenotype associations are lost, the different forms of RP can be divided into two main groups: i. rod-cone dystrophy (RCD) in which the rods are prevalently damaged (a RCD is diagnosed in about 80-90% of the total patients with RP and, thus, it can also be defined as typical RP); and ii. cone-rod dystrophy (CRD) in which the cones are mainly damaged (a CRD is diagnosed in about 10-20% of the total patients with RP and, thus, it can also be defined as atypical RP) [43, 44]. Several methods have been hitherto used to classify RPs:

- electroretinogram (ERG) to discriminate whether the alterations of the scotopic system (rods) predominate over those of the photopic one (cones) or vice versa;
- ophthalmoscopic retinal appearance (fundus oculi) to basically distinguish the localization and/or the characteristics of those peculiar degenerative changes observable in retina and retinal pigment epithelium (RPE), i.e. mid-peripheral or peripheral pigmentary deposits, sine pigment retinal aspect, retinitis punctata albescens with whitish spots, paravascular, sectorial, central or pericentral pigment deposits, and unilateral pigmentary lesions;
- mode of inheritance that includes monogenic autosomal dominant (AD), monogenic autosomal recessive (AR), monogenic dominant X-linked (XL) or, very rarely, monogenic recessive XL, digenic, and mitochondrial;
- age of onset that considers four possibilities for the beginning of specific RP symptoms and/or signs, detectable in children, adolescents, young adults or adults;
- molecular genetics that involves all the possible mutations causing the different RP phenotypes [6, 42].

These tapetum-retinal dystrophies are sometimes the epiphenomenon of a complex framework (syndromic RP in about 10-15% of cases), but more often they represent an isolated disorder (about 85-90 % of cases). In almost all cases, patients with syndromic RP are affected by a RCD, which usually resembles typical RP form. The more common RP-associated diseases are the following:

- Usher syndrome (type I, type II, type III, overall representing about 12% of RP), mainly related to neuro-sensorial hearing loss (secondary to disorders of cochlear cells) – the level of visual loss and, above all, hearing and balance loss depends on the Usher syndrome type;
- Bardet-Biedl syndrome, characterized by obesity, polydactyly, hypogonadism (especially in men), renal disorders and mental retardation;
- Laurence-Moon disease, characterized by mental retardation, hypogonadism, language impairment, ataxia, spastic paraplegia (without polydactyly, obesity, renal disorders);
- abetalipoproteinemia (Bassen-Kornzwig), characterized by lipoproteic dysmetabolism, malabsorption, progressive ataxic neuropathy, acanthocytosis;
- Alstrom disease, characterized by neurosensorial hearing loss, obesity, diabetes, dilated cardiomyopathy, chronic renal and epatic disorders;
- Cockayane syndrome, characterized by neurological disfunctions and some problems in growing;
- Refsum disease, characterized by lipoproteic dysmetabolism, peripheral neuropathy, ataxia, anosmia, ichthyosis and epiphyseal dysplasia;
- Friedreck ataxia, characterized by secondary ataxia to progressive central nervous system damages, growing and language disorders, cardiomyopathy;
- Kearns Sayre syndrome, characterized by external progressive ophthalmoplegia, heart disorders, cerebellar ataxia (occasionally with dystonia, myopathy, neurosensorial hearing loss, dementia, cataract, proximal tubular renal acidosis, diabetes, growth retardation, hypoparathyroidism);
- Mucopolysaccharidosis, characterized by bone abnormalities, mental retardation, corneal defects.

Usher syndrome, in which RP is associated with neuro-sensory deafness, represents the most frequent syndromic tapetum-retinal dystrophy [4, 8, 13, 15, 16]..

The phenotyping of both isolated and syndromic forms of RP can represent a very challenging task, that should be preferentially carried out in specialized referral Centers utilizing standardized diagnostic procedures and follow-ups. This clinical start-point is the necessary prerequisite to obtain an effectual collaboration among ophthalmologists, geneticists and epidemiologists, ideally articulated for a large-scale biobanking of patients inside each socio-sanitary catchment’s area. In fact, each patient with RP should be considered as a unique case on its own, in which the pathologic expressivity may be partially independent by the type of disease-gene but, at the same time and when it is possible, this patient should be also properly framed in well-defined genealogic and epidemiologic contexts. In other words, during the routine clinical practice of each referral RP Center, a standardized patient’s phenotyping and monitoring should be realized to maximize, in the mid- or long-term: the appropriateness of both epidemiological and bio-bank registers (involving the proband and, if applicable or possible, also his/her family tree), the chance of success of the multifaceted DNA analyses and, consequently, the cost/benefit ratio of these biomolecular investigations. The aforementioned collaborative study-attitude appears to be necessary to worldwide face the new challenges concerning both the molecular diagnoses and the gene therapies in RP patients. Considering the extraordinary variability of the disease-genes responsible for RP, as well as of their modifiers [6, 27-42], the applicability of these approaches will likely require the planning of several local, even if nationally or internationally coordinated, clinico-genetic studies targeted within each ethnic group.

## CLINICAL PHENOTYPING AND DIAGNOSTIC PROTOCOLS

The patients referred to a RP Center usually come to our observation for the following functional symptoms, ophthalmoscopic signs and/or visual field (VF) patterns:

- night-blindness (nyctalopia), variable photophobia, initial slight deterioration of quantity and/or quality of visual acuity (VA) – which are often distinctive symptoms of RCD;
- decrease in VA (often with reduction of fluent-reading), noteworthy photophobia, frequent dyschromatopsia, initial slight alteration of night-vision – which are often distinctive symptoms of CRD;
- rearrangement of the retinal pigment epithelium (RPE), variable attenuation of the retinal vessels, pigmentary deposits resembling bone spicules and/or various degrees of retinal atrophy (typically evident in the mid-peripheral sectors of the retina) possibly associated with pallor or waxy-pallor of the optic disc and normal looking macula or fine macular lesions – which are often distinctive signs of RCD;
- rearrangement of the RPE, pigmentary deposits resembling bone spicules and/or various degrees of retinal atrophy (typically evident in macular area and/or posterior pole of the retina) eventually anticipated by fine macular lesions and pallor of the optic disc, and then possibly associated with different levels of attenuation of the retinal vessels and macular degeneration – which are often distinctive signs of CRD;
- VF patterns characterized by patchy losses of peripheral vision evolving to ring shape scotoma with variable risk of tunnel vision – which are often distinctive of RCD;
- VF patterns characterized by central scotoma, then associated with variable patchy losses of peripheral vision – which are often distinctive of CRD.

Considering these specific, even if variably severe, eye disorders, the diagnostic protocols for patients with ascertained or suspected RP can be schematized in:

- basic protocol including those assessments strictly necessary for the diagnosis of RP
  - ophthalmologic examination (visual acuity, intraocular pressure, biomicroscopy of the ocular anterior segment and ophthalmoscopy of the ocular posterior segment)
  - visual field test (manual and/or computerized)
  - electroretinogram (full-field, scotopic, photopic, flicker and/or pattern)
  - genetic counseling
- full protocol including all those examinations useful for difficult differential diagnoses and/or complete patient’s phenotyping
  - ophthalmologic examination (visual acuity, intraocular pressure, biomicroscopy of the ocular anterior segment and ophthalmoscopy of the ocular posterior segment)
  - contrast sensitivity
  - color vision
  - visual field test (manual and/or computerized)
  - microperimetry
  - electroretinogram (full-field, scotopic, photopic, flicker and/or pattern)
  - micro-electroretinogram
  - multifocal electroretinogram
  - optical coherence tomography of the macular area
  - optical coherence tomography of the peri-papillary retinal nerve fiber layer
  - auto-fluorescent retinography
  - multifocal visual evoked potential
  - retino-choroidal angiography
  - genetic counseling
  - other particular, non-ophthalmologic, assessments necessary for the diagnosis of syndromic RP, such as audiometric and vestibular tests, olfactometric examination, renal echography, etc.

The evident complexity of this second protocol is further enhanced by the fact that for a consolidated diagnosis, also inclusive of an age-dependent RP staging of the disease’s expressivity, several patients should be monitored with periodical ophthalmologic checks for at least 24-36 months. The aspect of patient’s follow-up can become essential especially in the cases of RP diagnosed at an early stage of the disease; in fact, the prompt RP identification might represent the real turning-point on which are based current and, above all, future therapeutic possibilities for RP [45, 46]. Therefore, considering that RP can be transmitted with all the typologies of mendelian inheritance, each patient with RP should be managed like a potential proband by both ophthalmologist and geneticist, aiming to define the risk of disease or of non-affected-carrier status in the other members of the patient’s family.

## BASIC NOTIONS FOR GENETIC COUNSELING

The RP can be present in different individuals and/or in different generations of the same family. The inheritance can be direct and characterized by vertical transmission in the genealogic tree (autosomal dominant RP, AD-RP), or indirect and characterized by horizontal transmission in the genealogic tree (autosomal recessive RP, AR-RP). In several families, the disease can be inherited through the X chromosome (X-linked RP, XL-RP), where only males are affected if they receive the disease-gene from the mother, who is a non-affected carrier. If only one affected individual is present in a family and the available informations do not reveal any other case of RP in the genealogic tree, the disease should be labeled as sporadic RP. However, although in some cases these forms really begin in a normal family because the causal gene mutation occurs during the fetal development, in other cases they are wrongly defined as sporadic due to the loss or absence of disease’s informations in the past generations. Accurate ophthalmologic data of the proband and his/her relatives, together with a wide-ranging familial history, should be collected to efficiently guide the genetic counseling. This is a process by which patients and/or other family members, at risk of an inherited disorder such as RP, are advised about the typology and the possible consequences of the disorder, the probabilities of developing and/or transmitting it, and the options to prevent or treat it. This complex practice can be separated into two main aspects: diagnostic (the actual estimation of the risk) and supportive (the realistic explanation of the solutions).

The AD-RP typically has the following characteristics in the affected families: i. equal frequency in males and females; ii. vertical transmission of the disease-gene in the geologic tree; iii. procreative risk estimable around the 50%. According to this inheritance model, each patient with AD-RP should have one affected parent, as well other affected ancestors, such as one grandfather and/or one or more granduncles. However, this is just a theoretical model because, sometimes, misdiagnosed forms of RP do not allow to compose a reliable genealogic tree. Considering all forms of RP, the occurrence of AD disorder varies among the different ethnic groups, with an estimated average frequency approximately of 30-40% of cases [6, 42, 45]. In families with AR-RP, the disease usually appears with the following features: i. equal frequency in males and females; ii. horizontal presence of affected individuals in the geologic tree; iii. two non-affected carriers have a procreative risk estimable around the 25%. It is necessary that two healthy individuals, both carriers of one copy of the mutated gene, procreate to having an affected child. This situation occurs more frequently in small communities where the probability of parental consanguinity is higher. The usual lack of vertical transmission can often result in a difficult family tree drawing. Likewise the AD form, the occurrence of AR-RP changes among the different ethnic groups, with an estimated average frequency approximately of 50-60% of cases [6, 42, 45]. The XL-RP forms are characterized by: i. mutant gene is on the X chromosome and, thus, all men who inherit the causative mutation are diseased; ii. affected males transmit their defective X chromosome to all daughters, who are labeled as obligate non-affected carriers; iii. female carriers pass the defective X chromosome to half of their sons (who will be affected by RP) and to half of their daughters (who will be RP carriers too). Although each patient with XL-RP should have the mother as obligate non-affected carriers, in some families the misdiagnosing of this status does not allow to reliably compile the genealogic tree. The frequency of XL-RP is estimated around 5-15% of cases [6, 42, 45].

Although the most RP cases are monogenic, digenic and mitochondrial forms of RP have been described. In the complex context concerning the molecular genetics of patients with RP, the possibility to identify phenotype-genotype associations can be often frustrated by the existence of large genotypic diversity, unfeasibility in defining the inheritance model, racial variability of the causal gene mutations, inter- or intra-familial changeability of the phenotypic expressivity, and difficult clinical phenotyping of peculiar and/or advanced forms of RP [6-25, 27-42]. Despite the increasing possibilities of molecular diagnosis, at present approximately 30-50% of diagnosed RPs are attributable to genes that remain yet unidentified. Moreover, many genes for RP are causative of a small percentage of cases, with the exception of the rhodopsin gene (present in about 20-25% of AD-RP), USH2A gene (present in about 15-20% of AR-RP especially including many Usher syndrome type II), and RPGR gene (present in about 65-70% of XL-RP) [6, 42, 45]. RP genes code for molecules involved in different physiopathologic mechanisms, such as: phototransduction cascade, vitamin A metabolism, structural or cyto-skeletal functions, interactive cell-cell signaling or synaptic interaction, intron-splicing of RNA, intracellular trafficking, maintenance of cilia/ciliated cells, pH regulation, phagocytosis and so on; even so, the inventory of the mutations, ascertained or suspected, is constantly getting longer coming over 200 mutations present in more than 100 different genes [6, 42, 45].

## EPIDEMIOLOGY AND GENETICS

In the early 1980s, the overall rate of the numerous RP forms has been estimated in about 1 case per 3000-5000 inhabitants of the United States [7, 8]. Starting from 1984, in some districts of United States, United Kingdom, France, Spain and Germany, as well as in Norway, Denmark and Slovenia, several epidemiologic investigations on RP have been conducted in homogeneous populations of Caucasian ethnicity [9-13, 15-18, 21, 22, 25]. In particular, a study carried out in the Northern France revealed a prevalence of inherited retinal dystrophies equal to 1:2409 within a population of about 4 million people [25]. Exclusively considering the diagnoses of RP, in 2002 the estimated disease’s prevalence was 1:3943 among Danish population [17] whereas in Norway, fifteen years before, it had been rated 1:4440 [13]. More recently, a study carried out in the Southern Germany has documented that approximately 8% of the cases of blindness was caused by tapeto-retinal degeneration [22].

In other Countries, the available epidemiological data as regards of RP are still fragmentary and/or just partially accessible. In Veneto, a Region of the Northern Italy inhabited by about 4.9 million people, we have estimated that from 2006 to 2009 the official certifications of RP in the Rare Diseases Register are more than doubled. However, also the more recent estimation of RP prevalence, equal to about 1:8300, is still too much low in comparison with the data of the aforementioned European Countries [13, 17, 25]. On the other hand, exclusively considering the area where our specialized referral Center is activated from almost eight years (i.e. the Province of Padova inhabited by more than nine hundred thousand people), the certified RP diagnoses arrive to about 1 case per 5200 individuals. The considerable reduction of this epidemiologic and diagnostic gap indirectly emphasizes the importance of these peculiar clinico-rehabilitative Center, able to give a landmark for RP patients, to manage both probands and their relatives, to support the geneticists during their awkward biomolecular investigations. In addition, the effectual collaboration between referral RP Centers and Associations of RP patients should promote a proper socio-sanitary awareness about this disease, especially in those districts characterized by an increased RP occurrence due to high rate of consanguinity.

In view of the above-reported findings of RP prevalence in Caucasian populations [13, 17, 25], more than 3000 Italian families counting about 12000-15000 patients with inherited retinopathies should be hypothetically registered and classified. Although a part of these families (patients) have been hitherto analyzed for the several known mutations associated with RP, the causative genetic alteration has been discovered just in fewer than 45-50% of patients, with the exception of XL-RP forms for which this percentage can arrive to 65-75%. These limited results in molecular diagnosis resemble those obtained in other Western Countries suggesting, once again, that it is necessary a more systematic, diffused and comprehensive clinico-genetic management of patients with inherited retinal dystrophies, in a context of a RP-dedicated health network. Considering that, at present, these groups of diseases are just very partially curable, molecular diagnosis represents one of the most relevant information for the patients suffering from RP, as well as for their families. In Mendelian diseases, such as the various forms of RP, the gene identification is traditionally based on two approaches: i. positional cloning of regions specifically linked to the disease by both linkage analysis and homozygosis mapping; ii. functional cloning of the genes involved in the pathogenetic mechanisms of the disease. However, during the last years, the use of high-resolution genome-wide arrays has led to the characterization of several causative RP-associated genetic loci. Expectantly, the next widespread availability of new-generation sequencing machines (adapted for the clinical employment) will allow to simultaneously analyze all the genes associated with RP or, if certainly established the inheritance model (AD, AR or XL), all the genes associated with that specific hereditary pattern of RP. With the exploitation of these lab technologies, the molecular diagnostic tests of a RP patient and of his/her relatives may be carried out in one or just few months rather than one year or more, effectively supporting the advancement of an essential final necessity, i.e. the molecular classification of the RPs [6, 42]. In health and scientific fields, all the research programs aimed to more closely connect large database of RP cases or pedigrees with genetic studies on these groups of disorders are warranted to: i. take real clinical advantage of the high quantitative efficiency of the latest diagnostic methods regarding the already known RP mutations; ii. characterize novel disease-genes associated with the numerous molecular forms of RP. In this way, it will be finally possible to give concrete diagnostic answers to a considerable part of RP patients starting from a large-scale attitude of good epidemiologic practice to arrive at a factual good clinical practice, mainly composed of proper phenotyping and biobanking. As recently indicated by the proponents of the European Glaucoma Society GlaucoGENE project, “The use of non-specific or poorly defined phenotypes may partly explain the limited progress so far in glaucoma complex genetics” [47]. Referring to the RPs, this statement sounds more relevant than ever. In fact, only a synchronous advancement of the epidemiologic, clinical and genetic knowledges on RP will consent to effectually bridge the current gap from the lab to the patients.

## CONCLUSIONS

In several Health Systems, the overall management of the RP is difficult also because some important epidemiological issues are still unresolved for the lack of a shared and organized RP network, which is unavoidably related to risk of: i. quantitative underestimation of the disease; ii. limited socio-sanitary utility of the informative data included in the disease’s register. In view of the complexities inherent to an all-embracing approach toward RPs, the lack of an adequate epidemiologic register can have very negative impact on the diagnostic, preventive, therapeutic, rehabilitative and psychological management of many patients suffering from these dramatic eye diseases. In particular, an appropriate framework of a modern RP-database does not leave aside some critical aspects – of course, respecting both the privacy of each registered person and the willingness to share the data by the Health structures belonging to the certification-system:

- standardization of the diagnostic protocols utilized for the ophthalmologic classification of the disease, also detailing, if any, the deficiencies and/or the weak-points of this phenotyping procedure;
- large share of the methodology employed for the taxonomy of the inherited tapeto-retinal dystrophies;
- standardization of the approach utilized to stage the disease’s expressivity by means of an age-dependent grading system;
- report on both the therapeutic plans and the rehabilitative approaches dedicated to each affected individual;
- accurate clinical informations about other possible eye disorders associated with the tapeto-retinal degeneration, such as keratoconus, glaucoma, cataract, cystoid macular edema, Coats-like exudative vasculopathy, retinal angiomatous proliferation, choroidal neovascularization and so on;
- non-ophthalmologic clinical informations about the syndromic forms of the disease;
- description of the genealogic tree providing, if any, for the connections among that specific registered patient, the other affected relatives with a certified diagnosis and/or the non-affected (healthy) carriers of the family;
- informative electronic and anonymous links with the registry offices to possibly trace the first ancestral proband of the disease-gene and his/her area of birth;
- informations about the biobanking of blood, DNA or other biologic materials of the patient (if happened);
- report on the causative gene mutation (if definitely recognized);
- report about the presence of intra-familial homogeneity or heterogeneity in disease’s expressivity;
- report on the potential modifiers of the disease’s expressivity (inherited or acquired);
- obligation of patient’s certification or patient’s forwarding to a certifying Center by the ophthalmologist who identifies an affected individual;
- no intersection between the epidemiologic registration and legal-medicine implications, ensuring both the anonymity for the patient and the professional secrecy for the certifying physician.

In the next future, the deficiency in each of the above-listed points can represent a negative aspect, potentially able to counteract the good clinical governance of severe rare diseases like RPs. Although the individuals with these neurodegenerative disorders represent a relatively small percentage in proportion to the general population, their socio-sanitary burden is undoubtedly of critical importance, especially considering the frequent early onset of the disease, the procreative risk of disease’s transmission and the almost total lack of effective therapeutic strategies.
